# Ocular Motor Function in Patients with Bilateral Vestibular Weakness

**Published:** 2016-05

**Authors:** Seyyed Amir Hossein Ghazizadeh Hashemi, Sadegh Jafarzadeh, Majid Haddadi Aval, Reza Hosseinabadi

**Affiliations:** 1 *Department of ENT, School of Medicine, Shahid Beheshti University of Medical Sciences, Tehran, Iran.*; 2 *Department of Audiology, School of Paramedical Sciences, Mashhad University of Medical Sciences, Mashhad, Iran.*; 3 *Department of Audiology, Tehran University of Medical Sciences, Tehran, Iran.*

**Keywords:** Gaze stability, Ocular motor, Vertigo.

## Abstract

**Introduction:**

Patients with bilateral weakness (BW) have many difficulties in gaze stability that interfere with their normal function. The aim of this study was to evaluate ocular motor functions in patients with BW to better understand the problem of gaze instability in these patients.

**Materials and Methods:**

Patients were referred from the Otolaryngology Department for Vestibular Assessment to our clinic between November 2014 and March 2015. We assessed ocular motor function (gaze, saccade, and smooth pursuit) in patients over the age of 18 years with BW, as verified by a caloric test.

**Results:**

Seventy-eight patients completed all the tests. The mean age of patients was 51.9 (±15.9) years, and 47 (60%) were female. Abnormal results were found in five (6.4%), 32 (41%), and seven (9%) patients with respect to gaze, smooth pursuit, and saccade, respectively. There were positive but relatively weak relationships between age and ocular motor results.

**Conclusion:**

Patients with BW suffer from dizziness and unsteadiness. These patients have abnormal function in ocular motor (especially smooth pursuit) tests. The ocular motor dysfunction is responsible for gaze instability in static positions such as standing.

## Introduction

Bilateral weakness (BW) refers to bilateral and abnormally low function of the horizontal semicircular canals, superior vestibular nerve, and vestibular nuclei in a caloric test. It is a common sequela of vestibular disorders such as Meniere’s disease, vascular insufficiency, vestibular ototoxicity, and autoimmune disease. Patients with BW experience many problems such as vertigo, imbalance, and dynamic symptoms such as vestibular ocular reflex (VOR) dysfunction, gaze instability, and visual blurring due to oscillopsia. Visual blurring occurs because of deficient VOR during head movement such that the patient relies on compensatory catch-up saccade (1). This situation may occur during reading, walking (2), driving, or any other situation that requires head movement (3).

However, some patients with BW have difficulties in gaze stability even without head movement. The ocular motor abnormality (gaze, saccade, and smooth pursuit) represents abnormal function of the central vestibular system and gaze instability for static and moving objects (4). These tests have a major role in detecting vestibular disorders such as acute vestibular syndrome and differential diagnosis between central and peripheral lesions (5), as well as abnormal results of ocular motor function observed in many vestibular disorders (6).

The aim of this study was to evaluate ocular motor function in patients with bilateral weakness in order to better understand gaze instability problems in these patients, and to use this information for vestibular rehabilitation.

Additionally, because of the dependency of ocular motor function on the visual system and patient’s age (7-9), we also measured the relationship between the aging process and ocular motor function that was not related to visual problems. 

## Materials and Methods

Subjects were referred from the Otolaryngology Department for Vestibular Assessment to our clinic between November 2014 and March 2015. All subjects had vertigo and/or unsteadiness and vestibular disorders including Meniere’s disease, vestibular neuritis, and head trauma, as diagnosed by an otolaryngologist. We performed routine vestibular assessment using electronystagmography (Hortmann, Otometrics, Denmark) for each patient.

The inclusion criteria were age above 18 years and BW in the caloric test (slow phase velocity lower than 12°/s for summation of warm and cold irrigation on each side) (10,11). We excluded all patients with middle-ear abnormalities such as tympanic membrane perforation (confirmed by otoscopy, tympanometry and audiometry), visual disorders (such as cataract) and history of ear or eye abnormality or surgery.

All patients were informed about the study process and signed an informed consent form prior to any tests.

*Procedure*

Otoscopy (Reister, Germany), audiometry (Madsen, Denmark), and 226-Hz probe tone tympanometry (Madsen, Denmark) were performed to rule out middle-ear abnormalities. In the audiometry assessment, patients responded to air conduction and bone conduction stimuli in a sound-treated room using a modified Hughson-Westlake procedure. 

Patients were requested not to eat for 6 hours prior to electronystagmography (ENG) testing and to suspend using vestibular suppressant drugs such as Betaserc or any other medicine prescribed for vertigo for 2 days prior to the tests. 

The ENG test was performed according to standard protocols for a two-canal recording of horizontal and vertical eye movements. The skin was cleaned and the standard cup electrodes placed on the outer canthus of the eyes for the horizontal canal and up and down of the eye for the vertical canal. Ocular motor tests (gaze, smooth pursuit and saccade) were performed using standard ENG computerized settings, according to the standard protocol (Hortmann, Otometrics, Denmark). Evaluation of optokinetic function requires 90% filling of visual field with stimulus, which is not possible using the ENG/VNG setting. Therefore, in many clinical settings (including our study), this test is not performed. 

*Data analysis*

Data were analyzed using SPSS 19.0 software. Descriptive analysis was used to define the proportion of ocular motor dysfunction in BW patients and the mean (and standard deviation) patient age. Pearson correlation was used to determine the relationship between ocular motor results and age. 

## Results

*Patients*

In total, 105 people entered the study, although 27 were subsequently excluded. The mean age of the remaining 78 participants was 51.9 (±15.9) years, and 47 (60%) were female. Abnormal results were found in five (6.4%), 32 (41%) and seven (9%) patients with respect to gaze, smooth pursuit and saccade, respectively. This shows that BW patients have greater difficulty in following objects.Table 1 shows the correlation between age with gaze, smooth pursuit and saccade results.

**Table1:** The relationship between age and abnormal ocular motor responses

		**Gaze**	**Smooth pursuit**	**Saccade**
All patients	Correlation	0.272*	0.519*	0.307*
	P value	0.016	0.000	0.006
Up to 40 years old.	Correlation	0.109	0.160	0.569*
	P value	0.597	0.435	0.002
Older than 40 years.	Correlation	0.366*	0.469*	0.408*
	P value	0.008	0.000	0.003

* - correlation is significant at the 0.05 level.

The abnormal ocular motor function results had a positive but weak relationship with patients’ age. However, this finding is not sufficient to assume that the aging process is a cause of ocular motor abnormalities.

## Discussion

The dynamic visual acuity test usually emphasizes gaze stability in dynamic situations such as the turning of the head (12). These tests show gaze instability in different patients with vestibular disorders; however, our study shows that patients with BW have gaze instability even in static positions and when performing simple tasks such as following an object. Patients with BW tend to have more abnormal results in smooth pursuit rather than gaze or saccade, thus the greater difficulty in following a moving object. This may have serious consequences because in everyday activities, rather than a non-laboratory situation, smooth pursuit has a greater role in gaze stability, especially in self-generated movements (13). Ocular motor function and especially smooth pursuit results tend to be abnormal in chronic dizziness (6). We also observed positive, but relatively weak, relationships between ocular motor abnormalities and age. The aging process causes more abnormal results in ocular motor testing. Other studies have also shown similar results (7-9,14). This process usually begins in the fourth decade of life and results in reduced gain for smooth pursuit (7), increased saccade latencies (8), decreased saccade velocity (9), and even reduced saccadic function in the real word (14). However, the aging process is not responsible for all abnormal results in smooth pursuit, especially among younger individuals.

Different areas in the brain stem, central nervous system, and visual and vestibular pathways are responsible for normal ocular motor functions (15,16). Therefore, the abnormality in ocular motor function may stem from the visual or vestibular system (17). Postural control and Postural development are related to ocular motor function (18), and older patients usually develop imbalance and gait performance related to vestibular abnormality and gaze instability (19). By ruling out visual abnormality, we showed that ocular motor dysfunction, and particularly abnormal results of smooth pursuit, coexist with bilateral vestibular weakness; therefore, for diagnosis of vestibular disorders, a complete history and multiple examinations of eye movement are required (20).

BW affects gaze stability. Gaze instability in a static situation such as standing could result in an imbalance, and has serious adverse effects on patients’ quality of life (21). 

## Conclusion

Patients with BW suffer from dizziness and unsteadiness. These patients have abnormal function in ocular motor tests. Ocular motor dysfunction is responsible for gaze instability in static positions such as standing.
